# Functional assessments of *Psidium guajava* L. and *Morus alba* L. leaf extracts on postprandial glucose control

**DOI:** 10.1002/fsn3.4175

**Published:** 2024-04-23

**Authors:** Yen‐Ping Hsu, Wu‐Yuan Chen, Pao‐Chuan Hsieh, Yung‐Lin Chu

**Affiliations:** ^1^ Department of Food Science, College of Agriculture National Pingtung University of Science and Technology Neipu Pingtung Taiwan; ^2^ PD Biotech Co., Ltd Neipu Pingtung Taiwan; ^3^ Pingtung Christian Hospital Pingtung City Pingtung Taiwan

**Keywords:** diabetes, *Morus alba* L., *Psidium guajava* L., α‐glucosidase

## Abstract

Diabetes is a leading cause of death, according to statistics published by the Department of Health, Executive Yuan of Taiwan. In modern medicine, diabetes can be controlled using various medications; however, some drugs often have undesirable side effects. It therefore became a goal to find plant‐based material that can reduce glucose concentration in the blood while reducing the incidence of complications and not causing side effects. Alpha‐glucosidase inhibitors (AGIs) are effective glucose‐lowering medicines and are enzymes essential to carbohydrate digestion. Inhibition of α‐glucosidase leads to a delayed and reduced rise in postprandial blood glucose levels. This study evaluates the inhibitory effect of mixed extracts of *Psidium guajava* L. and *Morus alba* L. leaves on α‐glucosidase activity and postprandial hyperglycemia in normal and diabetic rats. The inhibition of α‐glucosidase activity was assayed in vitro. Half maximal inhibitory concentration **(**IC_50_) values of *Psidium guajava* L. and *Morus alba* L. were 2.25 and 0.1 mg/mL, respectively. The IC_50_ value of a commercial anti‐hyperglycemic agent (Glucobay) is 6.41 mg/mL. The IC_50_ value of a mixture of extracts of *Psidium guajava* L. and *Morus alba* L. was 0.07 mg/mL. In cytotoxicity tests, survival percentages and shape did not significantly affect the murine embryonic liver cell line (BNL CL.2) when treated with varying concentrations of mixture extracts for varying periods of time. In summary, *Psidium guajava* L. and *Morus alba* L. showed positive anti‐diabetes activity and suggested promising potential for alternative functional foods for diabetes mellitus (DM) patients.

## Graphical abstract

*Psidium guajava* L. and *Morus alba* L. leaf mixed extracts could control blood sugar, and it also evaluates the stability and safety assessment in this research.

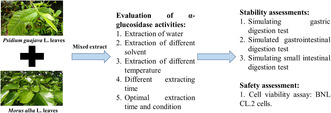

## 1 INTRODUCTION

Type 2 diabetes (T2D)‐related health expenditures are predicted to exceed 1054 billion USD in 2045. T2D leads to hyperglycemia and causes severe micro‐ and macrovascular complications, including atherosclerosis, cardiovascular disease (Sun et al., 2022), retinopathy, neuropathy, and nephropathy. Type 2 diabetes patients worldwide account for 95% of the diabetic population, primarily due to obesity, dietary habits, and a lack of exercise, which in turn leads to insulin resistance, resulting in elevated blood sugar levels as the body cannot effectively utilize the insulin (Botsi et al., 2023). Failure to effectively control and treat diabetes can lead to complications and an increased risk of heart disease, hypertension, and kidney problems, significantly burdening healthcare systems. There is no definitive cure for diabetes, so most patients opt for lifestyle changes, dietary adjustments, and medication therapy to manage the condition. However, long‐term use of commercially available medications can lead to side effects including weight gain, anemia, bloating, and diarrhea (Vestergaard, 2021). In response to this, over the past few years, researchers have increasingly sought effective ingredients in plant extracts to lower blood sugar levels for use in treating and preventing diabetes.

Alpha‐glucosidase is an exoenzyme found on the brush border of small intestine epithelial cells that hydrolyzes glucose molecules linked by α‐1,4 bonds at their non‐reducing ends (Lin et al., 2016). Enzymes responsible for the hydrolysis of oligosaccharides and glycosides, including sucrase, maltase, glucoamylase, and isomaltase, are collectively referred to as α‐glucosidases. Alpha‐glucosidase is the catalyst for the final step in carbohydrate digestion, breaking down ingested starch and oligosaccharides into monosaccharides for entry into the bloodstream and cellular utilization. Alpha‐glucosidase inhibitors work by reversibly inhibiting α‐amylase and competitively inhibiting small intestinal α‐glucosidase and hydrolytic enzymes (Yang et al., 2017). This inhibition hinders the breakdown and absorption of carbohydrates in the proximal intestine, releasing glucagon‐like peptide‐1 (GLP‐1) from L cells in the distal intestine. This, in turn, prolongs gastric emptying, suppresses appetite, enhances β‐cell sensitivity to glucose, and reduces postprandial blood sugar elevation and insulin levels (Kreitman et al., 2021).

This study investigated the inhibitory activity of *Psidium guajava* L. leaf and bark extracts on α‐glucosidase and their impact on blood sugar control. Previous studies showed that these compounds can control obesity and prevent the development of chronic cardiovascular diseases associated with obesity and diabetes (Beidokhti et al., 2020). Dr. Naowaboot's team tested a 50% ethanol extract of *Morus alba* L. leaves on Sprague–Dawley (SD) rats and ICR mice. They administered daily doses of 0.5 g and 1 g per kg of body weight of *Morus alba* L. leaf extract for 2 weeks (Lee et al., 2002). The blood sugar levels of diabetic rats decreased by 19 and 28%, respectively, compared to their blood sugar levels before being fed *Morus alba* L. leaf extract, demonstrating a significant change (Naowaboot et al., 2009).

Kimura et al. (2007) pointed out that *Morus alba* L. leaves contain 1‐deoxynojirimycin (DNJ), which has the potential to be developed as an α‐glucosidase inhibitor (AGI) to improve and prevent abnormal hyperglycemia associated with diabetes. Research confirmed that subjects who consumed *Morus alba* L. leaf powder containing 0.4 g, 0.8 g, and 1.2 g of DNJ effectively reduced postprandial blood sugar elevation. After continuously taking *Morus alba* L. leaf powder containing 1.2 g of DNJ for 38 days, their fasting blood sugar levels trended to decrease (Kimura et al., 2007). In recent years, an increasing number of scholars have been screening for substances with AGI properties among plant sources. In Mexico, 821 plant species have been classified as traditional herbal medicines, with 51 of them being used as antidiabetic agents (Ortiz‐Andrade et al., 2007). This highlights the growing importance of developing blood sugar‐lowering agents from plants.

In addition to being high in water content, guava contains proteins, fats, carbohydrates, and dietary fiber, as well as vitamins A, B, and C. Guava boasts the highest vitamin C content among fruits, with levels 8 to 9 times higher than oranges and 30 to 80 times higher than pineapples, bananas, tomatoes, and watermelons (Medina & Herrero, 2016). Guava also contains the minerals iron, calcium, and phosphorus, with the seeds being particularly rich in iron, a rarity among tropical fruits. Guava leaves contain volatile oils, quercetin, guaijaverin, wax, resin, sugars, essential oils, lower terpenes, flavonoids, tannins, triterpenoids, and steroids, among other compounds. Additionally, experimental evidence suggests that guava juice has a blood sugar‐regulating effect in mice (Cheng & Yang, 1983). The team of Dr. Andallu and Varadacharyulu investigated the effects of adding mulberry leaf powder of various concentrations to the diet of streptozotocin‐induced diabetic rats. Their findings showed that adding 25% *Morus alba* L. leaf powder to the diet led to a reduction in fasting blood sugar and improved control of urinary sugar levels after 8 weeks. This may be due to the inhibitory effects of *Morus alba* L. leaves on the activity of α‐glucosidase, α‐mannosidase, and β‐galactosidase (Andallu & Varadacharyulu, 2007). *Psidium guajava* L. leaves can also reduce lipid peroxidation and cholinesterase activity, increase superoxide dismutase activity, and reduce free radical production, thereby lowering the risk of gout, atherosclerosis, and other cardiovascular diseases (Irondi et al., 2016). They also studied the antioxidant potential of *Psidium guajava* L. leaves extracted using different solvents. In vitro, test results showed that a methanol extract of guava leaves had a high content of phenolic compounds, exhibited the ability to inhibit lipid oxidation, scavenge free radicals, and also demonstrated antioxidant properties (Camarena‐Tello et al., 2018). These results highlight the important role of phenolic compounds in the antioxidant qualities of *Psidium guajava* L. leaves.

This research may be the first study of mixed extracts of *Psidium guajava* L. and *Morus alba* L. leaves, suggesting additional effects of controlling blood sugar. Furthermore, it was the first research to assess the stability of mixed extracts in digesting systems and the safety of normal cells.

## 2 MATERIALS AND METHODS

The materials selected for this experiment were fresh leaves of guava (*Psidium guajava* L.) and leaves of mulberry (*Morus alba* L.), provided by farmers in Neipu Township, Pingtung County, Taiwan.

### 2.1 Preparation of a mixed extract of *Psidium guajava* L. leaves and *Morus alba* L. leaves

Fresh *Psidium guajava* L. leaves and *Morus alba* L. leaves were separately cleaned and then dried with hot air at 50°C. After drying, the leaves were ground into powder using a grinder and passed through a sieve. The extraction method was adapted and modified by Dr. Katsube (2006). After extraction, the samples were centrifuged at 9335×*g* for 10 min, and the supernatant was filtered through Whatman No.1 filter paper. The filtrate was collected and concentrated using a vacuum concentrator at 50°C. The extracts were then reconstituted to a concentration of 0.1 g/mL using dimethyl sulfoxide (DMSO) and further diluted to a concentration of 5 mg/mL with a 0.1 M phosphate buffer solution (pH 6.8). The inhibitory effect of each extract on α‐glucosidase activity was determined using the α‐glucosidase inhibition assay method.

### 2.2 Reagents

Methanol, ethyl acetate, acetone, n‐hexane, and pancreatin were purchased from Ching Ming Chemical Co., Ltd. (Kaohsiung, Taiwan). Intestinal acetone powder from rats, 4‐nitrophenyl α‐d‐glucopyranoside, dimethyl sulfoxide (DMSO), gallic acid, quercetin, pepsin, bile extract, sodium ammonium phosphate (NaHNH_4_PO_4_·4H_2_O), nicotinamide (NA), and sucrose were all obtained from Sigma‐Aldrich (St. Louis, MO, USA). Sodium phosphate 12 hydrate (Na_2_HPO_4_·12H_2_O), sodium dihydrogen phosphate (NaH_2_PO_4_·2H_2_O), sodium carbonate (Na_2_CO_3_), aluminum nitrate‐9‐hydrate (Al (NO_3_)_3_·9H_2_O), phenol, sodium hydroxide (NaOH), potassium phosphate (K_2_HPO_4_), and sodium chloride (NaCl) were purchased from Nacalai Tesque, Inc. (Osaka, Japan). Folin & Ciocalteu's phenol reagent was sourced from Merck (Darmstadt, Germany). Potassium acetate (C_2_H_3_KO_2_) was obtained from Riedel‐de Haën (Germany). Sodium hydrogen carbonate (NaHCO_3_), magnesium sulfate heptahydrate (MgSO_4_·7H_2_O), and citric acid monohydrate (C_6_H_8_O_7_·H_2_O) were purchased from Nacalai Tesque, Kyoto, Japan. Potassium chloride (KCl), HCl, and citric acid (C_6_H_8_O_7_) were procured from Union Chemical Works Ltd. (Hsinchu, Taiwan). Dulbecco's MEM (DMEM), fetal bovine serum (FBS), trypsin–EDTA, penicillin G/streptomycin, and trypan blue were obtained from BRL Gibco (Grand Island, NY, USA). Glucobay® tablets of 50 mg acarbose were purchased from Bayer Taiwan Co., Ltd. (Taipei, Taiwan).

### 2.3 Preparation of α‐glucosidase

In our study, we extracted 0.5 g of intestinal acetone powder from a rat, added 4.5 mL of a 0.1 M (pH 6.8) phosphate buffer solution, mixed well, placed the solution in an ice bath for 30 min, centrifuged at 4830×*g* for 20 min, and collected the supernatant as the enzyme solution. After aliquoting, the solution was stored at −20°C. The substrate ρ‐nitrophenyl α‐D‐glucopyranoside (PNP‐G) was prepared by mixing with a 0.1 M (pH 6.8) phosphate buffer solution to yield a 10 mM ρ‐Nitrophenyl α‐D‐glucopyranoside (PNP‐G) substrate solution, which was prepared on the day of the experiment. The enzyme activity measurement method of Oki et al. (1999) was used with slight modifications. We added 40 μL of α‐glucosidase solution to 100 μL of the extract (0.1 M phosphate buffer solution pH 6.8), mixed, and allowed to react at 37°C for 5 min. We then added 380 μL of PNP‐G (10 mM) and continued the reaction at 37°C for 15 min. Finally, we added 500 μL of 0.1 M Na_2_CO_3_ and mixed well to deactivate the enzyme and terminate the reaction. Absorbance was measured at 400 nm using a spectrophotometer (triplicate readings), representing the test group's absorbance value. The control group used a 0.1 M pH 6.8 phosphate buffer solution instead of the extract. The background group only measured the absorbance of the extract, so it only required the addition of the extract, with the enzyme and substrate replaced by buffer solution. These results derive the optimal extraction conditions for *Psidium guajava* L. leaves and *Morus alba* L. leaves. The *Psidium guajava* L. and *Morus alba* L. leaf extracts were mixed in different proportions and then prepared in a phosphate buffer solution (pH 6.8) at a concentration of 5 mg/mL. The impact of each different mixing ratio on α‐glucosidase activity was then determined using the method of inhibiting α‐glucosidase activity.

### 2.4 Simulated gastrointestinal digestion test

Preparation of gastric enzyme solution (“gastric fluid”): Dissolve 1 g of pepsin in 100 mL of 0.1 N HCl. Preparation of pancreatin‐bile suspension: In 100 mL of 0.1 M NaHCO_3_, dissolve 0.2 g of pancreatin and 1.2 g of bile extract (Forbes et al., 1989).

### 2.5 Simulating a gastric digestion test

After mixing *Psidium guajava* L. leaf extract and *Morus alba* L. leaf extract in the optimal ratio, prepare a mixed plant extract in a 0.1 M KCl‐HCl buffer (pH 2) at a 5 mg/mL concentration. Add 1 mL of the mixed plant extract to 100 μL of gastric enzyme solution (using an equivalent amount of distilled water for the control group). Agitate the reaction mixture at 37°C for 2, 4, and 6 h. Afterward, heat the mixture at 100°C for 10 min to terminate the reaction. Let it cool. This is the gastric digestion product. Adjust the pH to neutral with 1 N NaOH and measure the effect of the mixed plant extract on α‐glucosidase activity after reacting with the gastric enzyme solution (Forbes et al., 1989).

### 2.6 Simulating small intestinal digestion

Immediately following different digestion times for gastric digestion, add the gastric digestion product to 100 μL of pancreatin‐bile suspension (use distilled water instead for the control group). Agitate the reaction mixture at 37°C for 4 h. Afterward, heat the mixture at 100°C for 10 min to terminate the reaction. Let it cool. This is the small intestine digestion product. Measure the effect of the mixed plant extract on α‐glucosidase activity after reacting with the gastric enzyme solution and pancreatin‐bile suspension (Forbes et al., 1989).

### 2.7 Total carbohydrate content

Determining total carbohydrates was carried out following the method described in a previous study (Yousaf et al., 2021). The test employed the phenol‐sulfuric acid method, and carbohydrate content was measured using a spectrophotometer. Mix 200 μL of the sample with 200 μL of a 5% phenol solution, and then quickly add 1 mL of concentrated sulfuric acid. Shake the mixture well, let it stand for 30 min, and measure absorbance at 490 nm using a spectrophotometer. Prepare a standard curve using different glucose concentrations and convert this to sugar content (g/g) per gram of extract.

### 2.8 Total polyphenolic compound content

First, create a gallic acid standard curve. Prepare different concentrations of gallic acid/50% ethanol solutions as standards. Take 200 μL of various gallic acid/50% ethanol solutions, add 200 μL of Folin & Ciocalteu's phenol reagent, shake well, and let this stand in the dark for 3 min. Then, add 40 μL of a 10% Na_2_CO_3_ solution to stop the reaction. Shake the mixture every 10 min, and after 1 h of reaction in the dark, measure absorbance at 735 nm using a spectrophotometer. Prepare a standard curve with triplicate readings for each concentration. To determine the total polyphenolic compound content in the mixed plant extract, dilute the samples obtained after the extraction of pomegranate leaves and mulberry leaves under optimal conditions to a concentration of 1 mg/mL using a 50% ethanol solution. Take some operation as standards analysis, and shake the mixture every 10 min, and after 1 h of response in the dark, measure absorbance at 735 nm using a spectrophotometer. Perform triplicate measurements for each sample and calculate the milligrams of gallic acid per gram of extract based on the standard curve (He et al., 2022).

### 2.9 Total flavonoid content

Refer to the test method proposed by a previous study (Liu et al., 2022). First, create a standard curve by preparing different concentrations of quercetin solution. Take 0.5 mL and add this to 1.5 mL of 95% ethanol, then add 0.1 mL of 1 M aluminum nitrate, 0.1 mL of 1 M potassium acetate, and 2.8 mL of distilled water. Mix well and let this stand in the dark for 30 min, then measure absorbance at 415 nm using a spectrophotometer. Dilute the mixed plant extract to an appropriate concentration, and take 0.5 mL for the above measurement. Compare the results with the quercetin standard curve and calculate the flavonoid content in milligrams per gram of extract.

### 2.10 Cell viability assay (MTT test)

BNL CL.2 (mouse normal liver cell line BCRC 60180): Obtained from the Bioresource Collection and Research Center, Hsinchu, Taiwan. The cell viability assay was conducted following the MTT assay method proposed in a previous study (Huang et al., 2015). Cell concentration was adjusted to 1 × 10^5^ cells/mL with DMEM. 100 μL of cell suspension was then seeded in a 96‐well plate and cultured at 37°C in a CO_2_ incubator for 12 h. After cell attachment, the culture medium was removed, and cells were washed twice with phosphate‐buffered saline. Different concentrations of mixed plant extract (100 μL) were added, while the control group received DMEM with 0.1% DMSO instead. Cells were cultured for 24, 48, and 72 h in a CO_2_ incubator at 37°C. Cell growth was observed under an inverted microscope and photographed. Subsequently, 100 μL of MTT solution (concentration 2 mg/mL) was added, and the cells were incubated in the dark at 37°C in a CO_2_ incubator for 4 h. After centrifugation (100×*g*, 10 min), the supernatant was removed, and 50 μL of DMSO was added for 15 min. Absorbance at 570 nm was measured using an ELISA reader. The difference between the control and test groups was compared, and the cell survival percentage (%) of the sample group was calculated as follows: Sample group absorbance value/control group absorbance value × 100%.

### 2.11 Statistical analysis

All test data underwent analysis of variance (ANOVA) through the Statistical Analysis System (SAS) software, and differences between groups were assessed using Duncan's multiple range test (He et al., 2022).

## 3 RESULTS AND DISCUSSION

It is normal for blood sugar to rise after you eat and then fall again as the cells in your body take in the sugar from your blood to use for energy or to store for later. However, type 2 diabetes will show consistently high blood sugar levels after eating. Hence, minimizing the postprandial blood glucose spike is an important issue for normal people and type 2 *Diabetes mellitus* (DM) patients. In addition, there are some methods that can control postprandial glycemia, such as inhibition of the enzymes α‐glucosidase and α‐amylase. In previous studies, some herbal materials, probiotics, and food can help us control our postprandial glucose levels (Barros et al., 2021; Korkmaz & Dik, 2023; Kumar et al., 2021). In our study, we focus on *Psidium guajava* L. leaves and *Morus alba* L. leaves.

**FIGURE 1:**
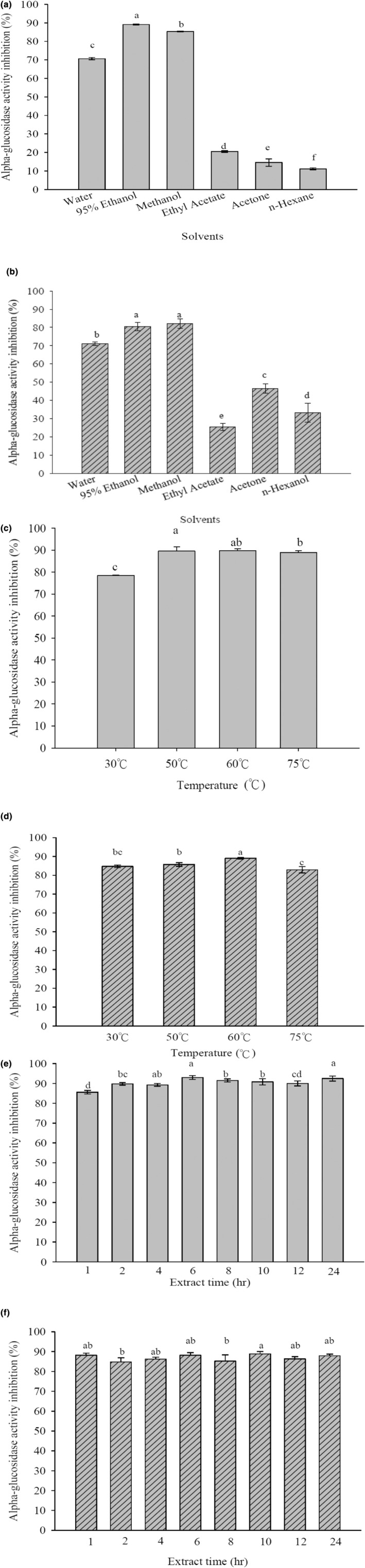
Inhibition of α‐glucosidase by extracts from *Psidium guajava* L. leaves prepared with different solvents (a). Inhibition of α‐glucosidase by extracts from *Morus alba* L. leaves prepared with different solvents (b). Inhibition of α‐glucosidase by ethanol extracts of *Psidium guajava* L. leaves extracted at different temperatures (c). Inhibition of α‐glucosidase by ethanol extracts from *Morus alba* L. leaves extracted at different temperatures (d). Inhibition of α‐glucosidase by ethanol extracts of *Psidium guajava* L. leaves extracted for different periods of time (e). Inhibition of α‐glucosidase by ethanol extract from *Morus alba* L. leaves extracted for different periods of time (f). Data are presented as the mean ± SD with statistically significant differences indicated by *p* < .05.

To investigate whether a mixture of plant extracts has a greater inhibitory effect on α‐glucosidase activity than single plant extracts, *Psidium guajava* L. leaves and *Morus alba* L. leaves were extracted under optimal conditions, and the powders were mixed in different proportions. These mixtures were prepared in a 5 mg/mL concentration with a phosphate buffer solution, and their effects on α‐glucosidase activity were determined using an inhibition method. *Psidium guajava* L. leaves and mulberry leaves were separately extracted using water, 95% ethanol, methanol, ethyl acetate, acetone, and hexane, employing solvents with varying polarities. The concentrated extracts were dissolved in DMSO to a concentration of 0.1 g/mL, and then each sample was diluted to a concentration of 5 mg/mL with a phosphate buffer solution. An in vitro assay was conducted to determine the optimal extraction solvent for each plant. The results are shown in Figure 1a,b.

From the results, it is evident that a 95% ethanol extraction of *Psidium guajava* L. leaves significantly outperformed other solvents in terms of enzyme inhibition, with an inhibition rate of 89.10 ± 0.21%. The inhibition rates for water, methanol, ethyl acetate, acetone, and hexane were 70.63 ± 0.63%, 85.34 ± 0.20%, 20.50 ± 0.47%, 14.54 ± 1.95%, and 11.14 ± 0.49%, respectively. On the other hand, mulberry leaves extracted with 95% ethanol and methanol exhibited better inhibition rates, with values of 80.41 ± 2.31% and 82.16 ± 2.50%, respectively. The inhibition rates for water, ethyl acetate, acetone, and hexane extraction were 71.10 ± 0.90%, 25.46 ± 2.06%, 46.56 ± 2.62%, and 33.19 ± 5.27%, respectively. These results indicate that the use of higher polarity solvents for extracting *Psidium guajava* L. leaves and mulberry leaves leads to higher enzyme inhibition, suggesting that the active substances responsible for inhibiting α‐glucosidase have higher polarity. However, considering the development of health food products for humans and animals, ethanol, which is safer for consumption, was chosen as the optimal extraction solvent for both *Psidium guajava* L. leaves and mulberry leaves. Therefore, subsequent experiments will use 95% ethanol as the extraction solvent to investigate the optimal extraction temperature for *Psidium guajava* L. leaves and *Morus alba* L. leaves.

To find the optimal extraction conditions for *Psidium guajava* L. leaves and *Morus alba* L. leaves using the chosen solvent, the effects of different temperatures and extraction times on enzyme activity were examined. The optimal extraction solvent for both *Psidium guajava* L. leaves and *Morus alba* L. leaves was determined to be ethanol. The boiling point of ethanol is 78.30°C. To avoid solvent evaporation during extraction, extraction was performed at 30, 50, 60, and 75°C for 1 h at each temperature. Samples were prepared using the method described above, and the inhibition rates of the different temperature extracts on enzyme activity were measured. The results are shown in Figure 1c,d.

From the results, it is clear that *Psidium guajava* L. leaves extracted with 95% ethanol at 50 and 60°C yielded better inhibition effects, with inhibition rates of 91.85 ± 2.25% and 89.78 ± 0.79%, respectively. The inhibition rates at 30 and 75°C were 78.43 ± 0.14% and 88.83 ± 0.98%, respectively. Similarly, for *Morus alba* L. leaves extracted with 95% ethanol, the best inhibition effect was achieved at 60°C, with an inhibition rate of 89.03 ± 0.43%. The inhibition rates at 30, 50, and 75°C were 84.73 ± 0.68%, 85.72 ± 0.98%, and 82.84 ± 1.70%, respectively. Since the difference in inhibition between 50 and 60°C for *Psidium guajava* L. leaves was minimal and traditional herbal remedies are often prepared at higher temperatures for better efficacy, 60°C was chosen as the ideal extraction temperature for both *Psidium guajava* L. leaves and *Morus alba* L. leaves. The investigation into the optimal extraction time continued.

Based on the experiments described above, it was determined that *Psidium guajava* L. leaves and *Morus alba* L. leaves, both extracted with 95% ethanol at 60°C, yielded the best inhibitory effects on enzyme activity. To identify the optimal extraction conditions for each plant, an investigation of the optimal extraction time was carried out. Extraction times of 1, 2, 4, 6, 8, 10, 12, and 24 h were examined for *Psidium guajava* L. leaves and *Morus alba* L. leaves extracted with 95% ethanol at 60°C. The results are shown in Figure 1e,f.

The results indicate that *Psidium guajava* L. leaves and *Morus alba* L. leaves extracted for different periods both achieved inhibition rates of over 80%. Therefore, the most energy‐efficient and cost‐effective extraction time was chosen, and 1 h was selected as the optimal extraction time, yielding inhibition rates of 87.70 ± 1.35% and 88.23 ± 0.99% for *Psidium guajava* L. leaves and *Morus alba* L. leaves, respectively.

**FIGURE 2:**
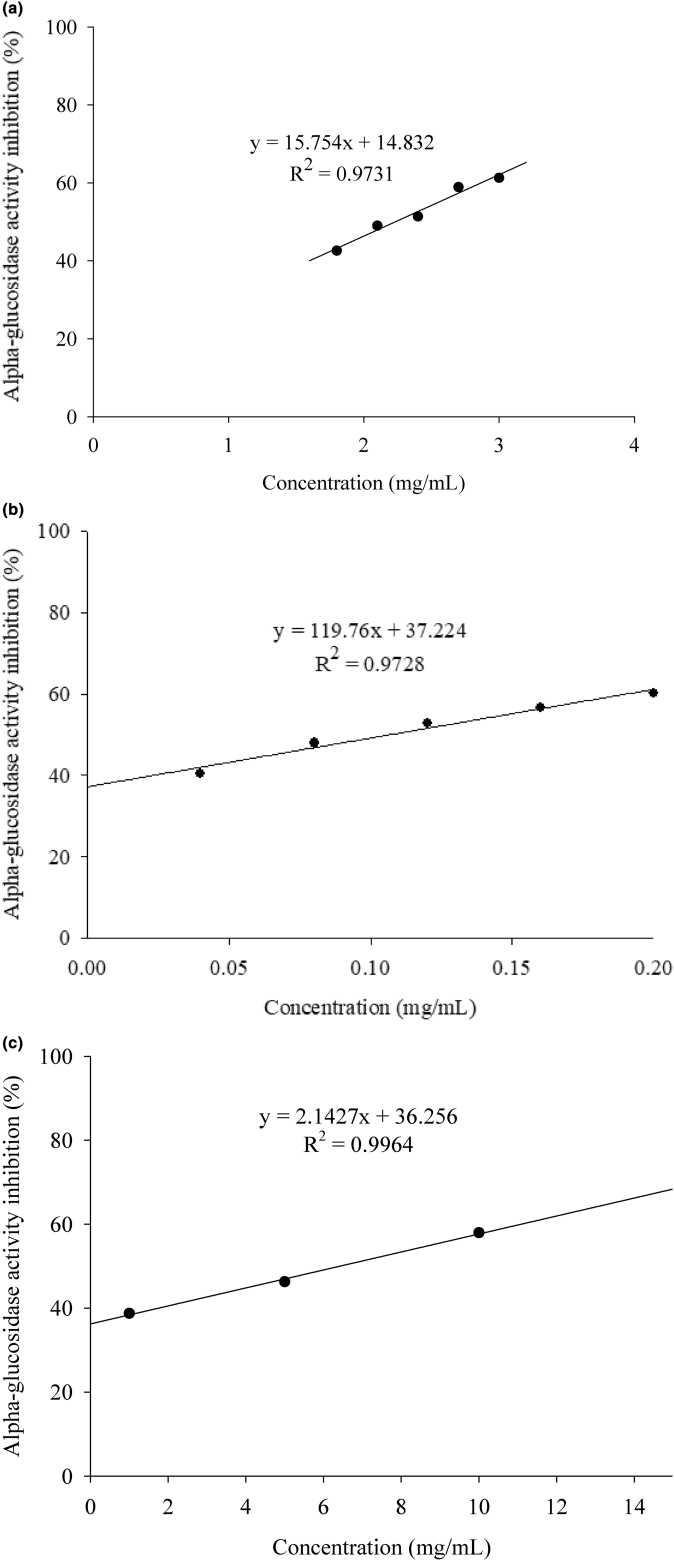
The IC_50_ value of *Psidium guajava* L. leaf extracts using optimal extraction procedures in inhibiting α‐glucosidase activity (a) The IC_50_ value of *Morus alba* L. leaf extracts by optimal extraction procedures in inhibiting α‐glucosidase activity (b) The IC_50_ value of Glucobay in inhibiting α‐glucosidase activity (c).

In summary, the optimal extraction parameters for *Psidium guajava* L. and *Morus alba* L. leaves are 95% ethanol extraction at 60°C for 1 h. The extracts obtained under these conditions were concentrated and freeze‐dried into powder form. The powders were then diluted to different concentrations with a buffer solution, and their inhibitory effects on α‐glucosidase activity were determined to calculate the half‐maximal inhibitory concentration (IC_50_) of the plant extracts, as shown in Figure 2a,b. The commercially available blood glucose‐lowering drug Glucobay contains acarbose as the active ingredient, with each tablet containing 50 mg of acarbose (Sun et al., 2014). The tablets were ground into powder, diluted to different concentrations with a buffer solution, and their inhibitory effects on enzyme activity were determined to calculate the IC_50_ of Glucobay, as shown in Figure 2c.

From these experimental results, it is clear that the IC_50_ values for *Psidium guajava* L. and *Morus alba* L. leaves extracted under optimal conditions were 2.25 and 0.1 mg/mL, respectively. In comparison, the IC_50_ value for Glucobay, the commercial blood glucose‐lowering drug, was 6.41 mg acarbose/mL.

**FIGURE 3:**
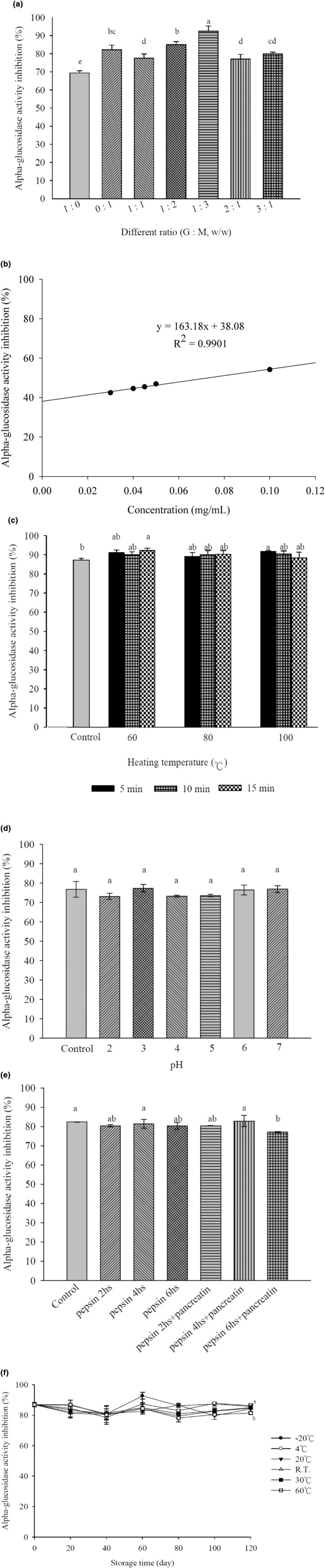
Inhibition of α‐glucosidase using mixture extracts of *Psidium guajava* L. and *Morus alba* L. leaves in different ratios (a) IC_50_ value for mixture extracts from *Psidium guajava* L. and *Morus alba* L. leaves in inhibiting α‐glucosidase activity (b). Inhibition of α‐glucosidase using mixture extracts of *Psidium guajava* L. and *Morus alba* L. leaves with various heating temperatures (c) Inhibition of α‐glucosidase using mixture extracts of *Psidium guajava* L. and *Morus alba* L. leaves with various pH (d) Inhibition of α‐glucosidase using mixture extracts of *Psidium guajava* L. and *Morus alba* L. leaves treated with pepsin and pancreatin (e). Effect of different storage conditions of mixture extracts of *Psidium guajava* L. and *Morus alba* L. leaves on the inhibition of α‐glucosidase (f). Data are presented as the mean ± SD with statistically significant differences indicated by *p* < .05.

To investigate whether a mixture of plant extracts has a greater inhibitory effect on α‐glucosidase activity than single plant extracts, *Psidium guajava* L. and *Morus alba* L. leaves were extracted under optimal conditions and mixed in different proportions. These mixtures were prepared in a 5 mg/mL concentration with a phosphate buffer solution, and their effects on α‐glucosidase activity were measured. The results are shown in Figure 3a. From the graph, it can be seen that the inhibition rate of the *Psidium guajava* L. leaf extract on α‐glucosidase was 69.36.

The above experimental results indicate the optimal ratio of *Psidium guajava* L. leaves and *Morus alba* L. leaves. After preparing the extract with this optimal mixing ratio, the solution was diluted into different concentrations using a buffer solution. Then, the inhibitory rate of the mixed plant extract on α‐glucosidase activity was measured using the α‐glucosidase inhibition method to calculate the IC_50_ of *Psidium guajava* L. and *Morus alba* L. leaves at the optimal mixing ratio, as shown in Figure 3b. From the experimental results, it is evident that the combination of *Psidium guajava* L. and *Morus alba* L. leaves has a synergistic inhibitory effect on α‐glucosidase activity, with an IC_50_ of 0.07 mg/mL, which is lower than that of the individual plant extracts of *Psidium guajava* L. leaves and *Morus alba* L. leaves. This experimental result also shows a lower IC_50_ than the commercially available blood glucose‐lowering drug Glucobay, indicating that this potential health food ingredient is worth further development and exploration.

Using the optimal extraction conditions to extract *Psidium guajava* L. and *Morus alba* L. leaves, it was found that a combination of *Psidium guajava* L. leaves and *Morus alba* L. leaves exhibited a synergistic effect when mixed in different proportions, resulting in an enhanced inhibitory effect on the enzyme compared to the individual plant extracts. The purpose of this study is to develop a beverage with blood glucose‐regulating effects in the future. Since the product development process requires sterilization before it can be marketed, further investigation is needed to understand the effect of the mixed plant extract on enzyme activity at different temperatures and heating times to assess its thermal stability, as shown in Figure 3c. The results in the figure indicate that when the mixed plant extract is heated at 60, 80, and 100°C for 5, 10, and 15 min, there is little difference in the impact on enzyme activity. The inhibition rates after heating at 60, 80, and 100°C for 15 min are 91.67 ± 0.42%, 90.37 ± 1.65%, and 88.36 ± 3.03%, respectively. These results suggest that the mixed plant extract has good thermal stability, and temperature variations and heating times do not significantly affect enzyme activity.

To understand whether the mixed plant extract is affected by pH during processing, the mixed plant extract was subjected to different pH conditions for 4 h. Then, the effect of the mixed plant extract on α‐glucosidase activity after different pH treatments was determined using the α‐glucosidase inhibition method. Phosphate buffer solutions with pH values ranging from 2.0 to 7.0 and citrate buffer solutions were prepared. The mixed plant extract was diluted into a 5 mg/mL extract using a phosphate buffer at pH 6.8, subjected to different pH conditions for 4 h, and then compared to a control group (using distilled water instead of buffer solutions with different pH values), as shown in Figure 3d. The results indicate that the mixed plant extract showed no significant difference in its effect on enzyme activity when subjected to conditions ranging from pH 2 to 7 for 4 h.

A simulated test of the human digestive tract was conducted, referring to the “Assessment Method for Promoting Iron Absorption Function of Health Foods” formulated by the Ministry of Health and Welfare (Forbes et al., 1989). This was done to understand whether the mixed plant extract loses its activity after being consumed by the human body due to the action of digestive enzymes. The extract was first dissolved in a buffer solution of 0.1 M KCl‐HCl (pH 2.0) and then subjected to agitation at 37°C for 2, 4, and 6 h after the addition of pepsin solution to simulate gastric digestion. The control group used distilled water instead of pepsin solution. A bile salt suspension was then added to the gastric digestion mixture and allowed to react for 4 more hours. Finally, the reaction was terminated by heating for 15 min. This process represented intestinal digestion. After cooling, the impact of simulated digestion on α‐glucosidase activity was measured. The results of the simulated gastrointestinal digestion test for the mixed plant extract are shown in Figure 3e. The figure shows that the control group had an inhibition rate of 82.31 ± 0.09% on the enzyme. After 2, 4, and 6 h of gastric digestion followed by 4 h of intestinal digestion, the inhibition rates were 80.30 ± 0.60%, 81.25 ± 2.26%, and 80.27 ± 1.80%, respectively, which were slightly lower than the control group by about 1–3%. In the case of intestinal digestion, after 6 h of gastric digestion followed by 4 h of intestinal digestion, the inhibition rate decreased by about 6% compared to the control group. These results suggest that pepsin and bile salts may slightly affect the mixed plant extract, but the solution still maintains an inhibition rate on enzyme activity of around 80%.

Freeze‐dried mixed plant extract was prepared into a highly concentrated extract with sterile water. After heating with steam at 100°C for 15 min, the solution was aliquoted into microcentrifuge tubes under a sterile hood and stored at different temperatures for 120 days, with samples taken every 20 days. Each sample was diluted to 5 mg/mL with a phosphate buffer (pH 6.8), and the effect of the mixed plant extract on α‐glucosidase activity was measured. The results are shown in Figure 3f. This figure indicates that under different storage conditions, the inhibitory activity of the extract on α‐glucosidase gradually decreased with time, but the decrease was not significant. Statistical analysis showed significant differences only between the samples stored at 60°C on day 0 and day 120, while there were no significant differences in inhibitory activity for the other storage conditions between day 0 and day 120. The results suggest that when stored at −20 and 4°C, the mixed plant extract can maintain an inhibition rate of over 85% after 120 days, indicating that the inhibitory capacity of the mixed plant extract on α‐glucosidase is not significantly affected by temperature and time. The experimental results align with the findings of Dr. Saeedeh (Arabshahi‐Delouee & Urooj, 2007). which showed that methanol extracts of mulberry leaves stored at 5°C for 30 days did not exhibit significant differences in antioxidant activity compared to day 0 but began to decrease with prolonged storage.

**FIGURE 4:**
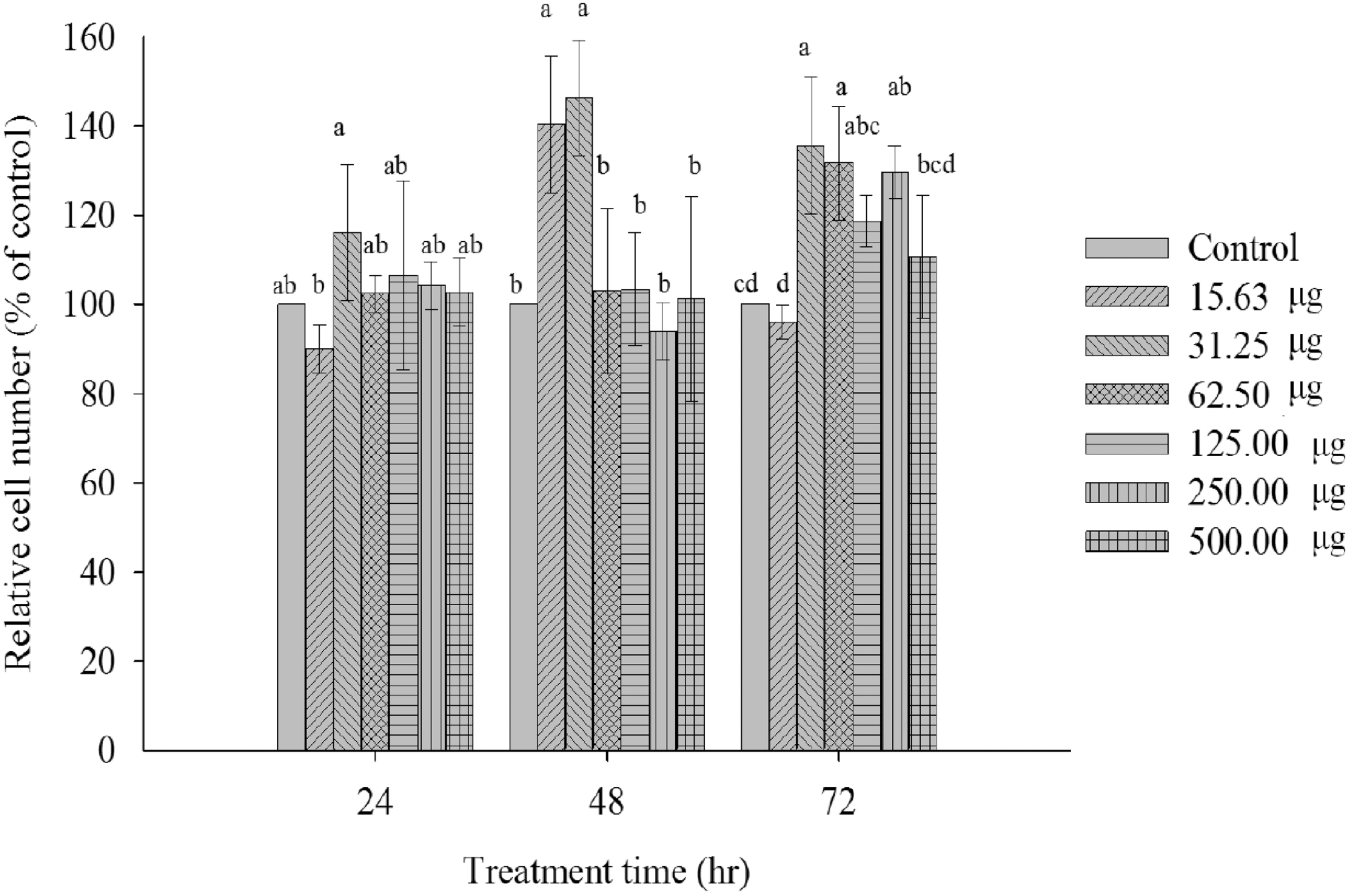
Effect of mixture extracts of *Psidium guajava* L. and *M. alba* L. leaves on the survival rate of BNL CL.2. Data are presented as the mean ± SD with statistically significant differences indicated by *p* < .05.

**FIGURE 5:**
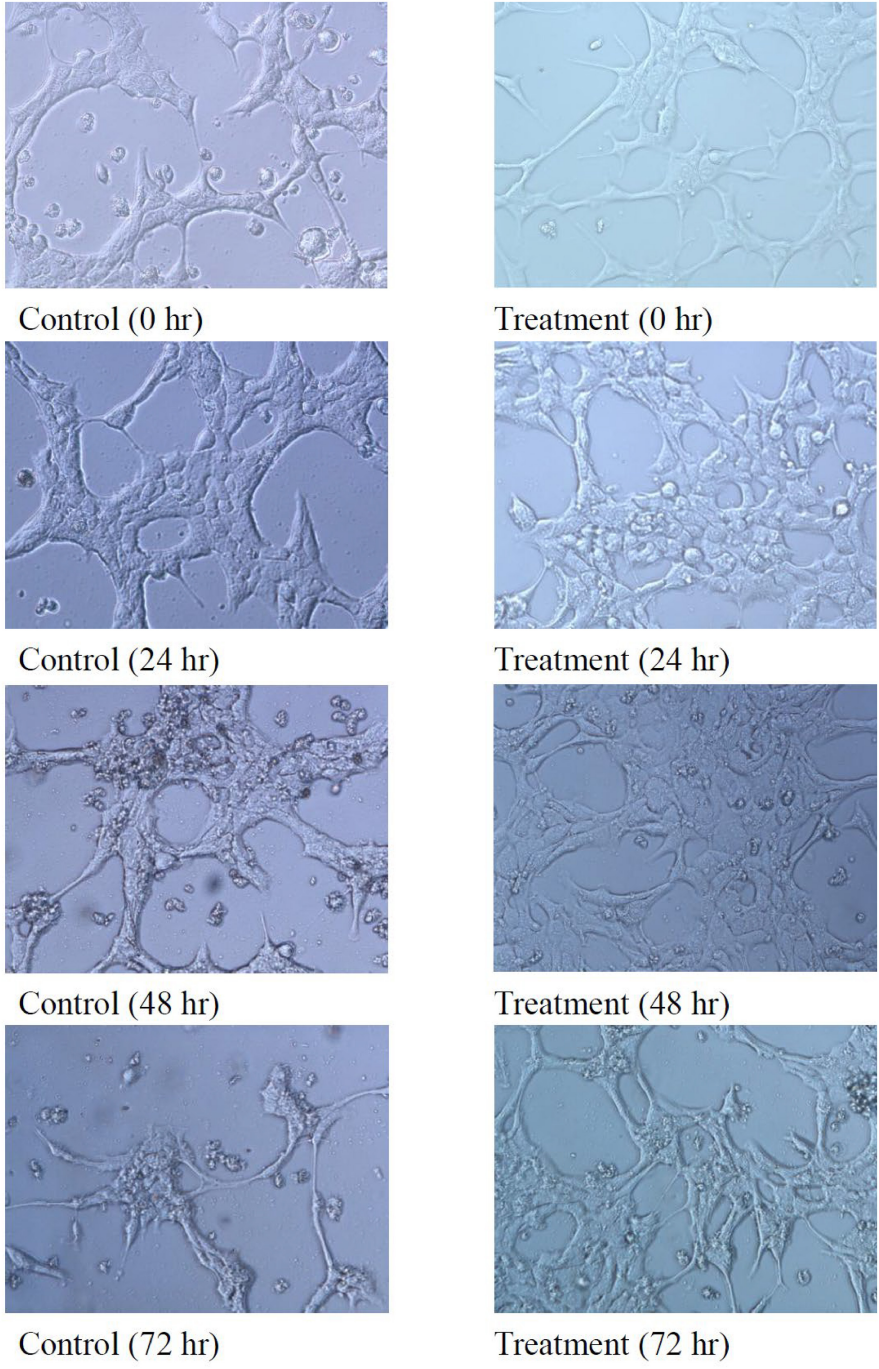
Morphology of BNL CL.2 treated with 500 μg/mL mixture extracts (200×).

**FIGURE 6:**
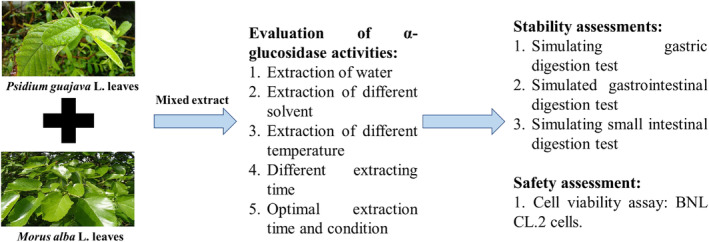
Graphic of content.

The method for determining cell viability was conducted using an MTT assay. Its primary principle involves utilizing an enzyme within the mitochondria to convert the soluble MTT tetrazolium from yellow into an insoluble purple crystal product. Subsequently, a solvent is used to dissolve the crystal product, and absorbance is measured at a wavelength of 570 nm using an ELISA reader. The amount of product formed is directly proportional to cell viability, with higher absorbance values indicating higher cell viability. Therefore, measuring the absorbance values after the reaction allows for the inference of cell viability. The experimental results are shown in Figure 4. As depicted in the figure, normal liver cells exposed to a low dose of the mixed plant extract for 24 and 48 h show an increase in cell growth. However, when exposed for 72 h, cell viability decreases, possibly due to the inhibition of cell growth caused by high cell density, leading to a reduction in cell numbers. Even at the highest concentration of the mixed plant extract (500 μg/mL), cell viability remains at 100%, indicating that the mixed plant extract is non‐toxic to normal mouse liver cells (BNL CL.2). Observations of cell morphology using an inverted microscope are presented in Figure 5. The results show that the mixed plant extract (500 μg/mL) does not induce any changes in cell morphology over different durations of exposure to normal liver cells. Interestingly, the cell count appears to be higher in cells exposed to the mixed extract (500 μg/mL) compared to the control group. In summary, these findings align with Zhang's research on the effects of polyphenol extracts from *Morus alba* L. in vivo (Zhang et al., 2009). The results indicate that polyphenols and flavonoids have no toxicity on liver cells (BNL CL.2) and may enhance the recovery of mitochondrial membrane potential, thereby protecting liver cells (Figure 6). The results indicate that polyphenols and flavonoids have no toxicity on liver cells (BNL CL.2) and may enhance the recovery cell viability, thereby protecting liver cells (Figure 4 and Figure 5).

The total carbohydrate content of the *Psidium guajava* L. and *Morus alba* L. leaf extracts obtained under optimal conditions was determined using the phenol‐sulfuric acid method for colorimetric analysis. The results of the total carbohydrate content in the *Psidium guajava* L. and *Morus alba* L. leaf extracts obtained under optimal conditions are presented in Table 1. The results show that the total carbohydrate content in *Psidium guajava* L. leaves is 27.58 ± 0.60%, while in mulberry leaves, it is 34.76 ± 1.90%. Literature suggests that polysaccharides in mulberry leaves can promote insulin release, increase glucose utilization in peripheral tissues, improve abnormal sugar metabolism, and consequently reduce blood sugar levels (Shen et al., 2008).

**TABLE 1:** Total sugar, total phenol, and flavonoid content of *Psidium guajava* L. and *Morus alba* L. leaf extracts.

	*Psidium guajava* L.	*Morus alba* L.
Total sugar (g/g)	0.28 ± 0.01^b^	0.35 ± 0.02^a^
Total phenols (mg/g)	130.83 ± 0.07^a^	43.50 ± 2.08^b^
Flavonoids (mg/g)	75.68 ± 3.28^a^	17.30 ± 0.54^b^

*Note*: Data are presented as the mean ± SD with statistically significant differences indicated by *p* < .05.

The total phenolic content of *Psidium guajava* L. and *Morus alba* L. leaf extracts obtained under optimal conditions was determined by reacting them with gallic acid and Folin & Ciocalteu's phenol reagent and measuring absorbance at 735 nm. Higher absorbance values indicate a greater amount of total phenolic compounds in the sample. The total phenolic content of *Psidium guajava* L. and *Morus alba* L. leaf extracts obtained under optimal conditions is shown in Table 1. The results reveal that the total phenolic content of *Psidium guajava* L. leaf extract is 130.83 ± 0.07 mg/g, while in *Morus alba* L. leaf extract, it is 43.50 ± 2.08 mg/g. Previous studies have shown that *Psidium guajava* L. leaves contain phenolic compounds with antioxidant properties (Chen & Yen, 2007). Previous studies also reported the presence of various phenolic compounds in *Psidium guajava* L. leaves, including tannins, polyphenolic compounds, flavonoids, pentacyclic triterpenoids, guajavaerin, and quercetin, all of which have antioxidant effects and may contribute to the beneficial effects of *Psidium guajava* L. leaf juice on blood sugar levels (Ojewole, 2005).

Flavonoids are natural antioxidants known for their ability to scavenge free radicals, remove reactive oxygen species, and reduce the oxidation of vitamins C and E. Flavonoids consist of different subgroups with varying physiological activities (Shen et al., 2022). The flavonoid content of *Psidium guajava* L. and *Morus alba* L. leaf extracts obtained under optimal conditions was determined and is shown in Table 1. The results indicate that the flavonoid content in *Psidium guajava* L. leaf extract is 75.68 ± 3.28 mg/g, while in *Morus alba* L. leaf extract, it is 17.30 ± 0.54 mg/g. Katsube et al. reported high flavonoid content, particularly quercetin (9 mg/g), in *Morus alba* L. leaf extracts obtained using 60% ethanol (Katsube et al., 2006). The results also suggest that flavonoid‐rich *Morus alba* L. leaf extracts have antioxidant activity and can delay the oxidation of low‐density lipoprotein (LDL), potentially preventing the development of atherosclerosis. High levels of glucose in the blood of diabetic patients can lead to the formation of advanced glycation end products (AGEs), which are closely associated with cardiovascular diseases. Wu et al. found that water extracts of *Psidium guajava* L. leaves containing quercetin at 5.30 ± 0.70 mg/g could inhibit AGE formation in vitro, reducing the risk of related diseases (Wu et al., 2009).

## 4 CONCLUSION

Our research has shown that *Psidium guajava* L. and *Morus alba* L. leaf extracts can inhibit α‐glucosidase activity (Figure 6). However, *Psidium guajava* L. leaves and *Morus alba* L. leaves obtained using 95% ethanol and extracted at 60°C for 1 h exhibit superior inhibitory activity against α‐glucosidase. Under optimal conditions, the half‐maximal inhibitory concentration (IC_50_) of the extracts on α‐glucosidase is 2.25 mg/mL for *Psidium guajava* L. leaves and 0.1 mg/mL for *Morus alba* L. leaves. In comparison, the IC_50_ of the commercially available blood‐glucose‐lowering drug (Glucobay) is 6.41 mg/mL, while the IC_50_ of the mixed plant extract is reduced to 0.07 mg/mL. Under the optimal extraction conditions of *Psidium guajava* L. and *Morus alba* L. leaf mixtures, the total carbohydrate content is 0.28 ± 0.01 g/g for *Psidium guajava* L. leaves and 0.35 ± 0.02 g/g for *Morus alba* L. leaves. Total phenolic content is 130.83 ± 0.07 mg/g for the *Psidium guajava* L. leaves and 43.5 ± 2.08 mg/g for the *Morus alba* L. leaves. Flavonoid content is 75.68 ± 3.28 mg/g for *Psidium guajava* L. leaves and 17.30 ± 0.54 mg/g for *Morus alba* L. leaves. In cell toxicity assays, when different concentrations of the mixed plant extract were added and incubated with normal liver cells (BNL CL.2) for 24, 48, and 72 h, there was no observed toxicity in terms of cell viability and morphology.

## AUTHOR CONTRIBUTIONS

**Yen‐Ping Hsu:** Data curation (lead); formal analysis (lead). **Wu‐Yuan Chen:** Funding acquisition (lead); investigation (supporting). **Pao‐Chuan Hsieh:** Conceptualization (lead); resources (equal); supervision (equal). **Yung‐Lin Chu:** Conceptualization (equal); supervision (lead); visualization (lead); writing – original draft (lead).

## CONFLICT OF INTEREST STATEMENT

The authors declare no competing financial interests.

## Data Availability

Data are openly available in a public repository that issues datasets with DOIs.

## References

[fsn34175-bib-0030] Andallu, B. , & Varadacharyulu, N. C. (2007). Gluconeogenic substrates and hepatic gluconeogenic enzymes in streptozotocin‐diabetic rats: Effect of mulberry (*Morus indica* L.) leaves. Journal of Medicinal Food, 10(1), 41–48. 10.1089/jmf.2005.034 17472465

[fsn34175-bib-0034] Arabshahi‐Delouee, S. , & Urooj, A. (2007). Antioxidant properties of various solvent extracts of mulberry (*Morus indica* L.) leaves. Food Chemistry, 102(4), 1233–1240. 10.1016/j.foodchem.2006.07.013

[fsn34175-bib-0031] Barros, C. P. , Grom, L. C. , Guimaraes, J. T. , Balthazar, C. F. , Rocha, R. S. , Silva, R. , Almada, C. N. , Pimentel, T. C. , Venâncio, E. L. , Collopy Junior, I. , PMC, M. , Freitas, M. Q. , Esmerino, E. A. , Silva, M. C. , Duarte, M. C. K. H. , Sant'Ana, A. S. , & Cruz, A. G. (2021). Paraprobiotic obtained by ohmic heating added in whey‐grape juice drink is effective to control postprandial glycemia in healthy adults. Food Research International, 140, 109905. 10.1016/j.foodres.2020.109905 33648206

[fsn34175-bib-0001] Beidokhti, M. N. , Eid, H. M. , Villavicencio, M. L. S. , Jäger, A. K. , Lobbens, E. S. , Rasoanaivo, P. R. , McNair, L. , Haddad, P. S. , & Staerk, D. (2020). Evaluation of the antidiabetic potential of *Psidium guajava* L. (Myrtaceae) using assays for alpha‐glucosidase, alpha‐amylase, muscle glucose uptake, liver glucose production, and triglyceride accumulation in adipocytes. Journal of Ethnopharmacology, 257, 112877. 10.1016/j.jep.2020.112877 32305639

[fsn34175-bib-0002] Botsi, E. , Karatzi, K. , Mavrogianni, C. , Tsochev, K. , González‐Gil, E. M. , Radó, S. , Kivelä, J. , Wikström, K. , Cardon, G. , Rurik, I. , Liatis, S. , Tankova, T. , Iotova, V. , Moreno, L. A. , Makrillakis, K. , Manios, Y. , & Tsigos, C. (2023). Association of diet quality with glycemia, insulinemia, and insulin resistance in families at high risk for type 2 diabetes mellitus in Europe: Feel4 diabetes study. Nutrition, 105, 111805. 10.1016/j.nut.2022.111805 36335874

[fsn34175-bib-0003] Camarena‐Tello, J. C. , Martínez‐Flores, H. E. , Garnica‐Romo, M. G. , Padilla‐Ramírez, J. S. , Saavedra‐Molina, A. , Alvarez‐Cortes, O. , Bartolomé‐Camacho, M. C. , & Rodiles‐López, J. O. (2018). Quantification of phenolic compounds and in vitro radical scavenging abilities with leaf extracts from two varieties of *Psidium guajava* L. Antioxidants (Basel), 7(3), 34. 10.3390/antiox7030034 29495514 PMC5874520

[fsn34175-bib-0004] Chen, H. Y. , & Yen, G. C. (2007). Antioxidant activity and free radical‐scavenging capacity of extracts from guava (*Psidium guajava* L.) leaves. Food Chemistry, 101(2), 686–694. 10.1016/j.foodchem.2006.02.047

[fsn34175-bib-0005] Cheng, J. T. , & Yang, R. S. (1983). Hypoglycemic effect of guava juice in mice and human subjects. The American Journal of Chinese Medicine, 11(1–4), 74–76. 10.1142/S0192415X83000124 6660217

[fsn34175-bib-0006] Forbes, A. L. , Arnaud, M. J. , Chichester, C. O. , Cook, J. D. , Harrison, B. N. , Hurrell, R. F. , Kahn, S. G. , Morris, E. R. , Tanner, J. T. , & Whittaker, P. (1989). Comparison of in vitro, animal, and clinical determinations of iron bioavailability: International nutritional anemia consultative group task force report on iron bioavailability. The American Journal of Clinical Nutrition, 49(2), 225–238. 10.1093/ajcn/49.2.225 2644802

[fsn34175-bib-0007] He, J. J. , Chiu, C. H. , Gavahian, M. , Ho, C. T. , & Chu, Y. L. (2022). Development and application of edible coating on dried pineapple exposed to electrical blanching. Journal of Food Processing and Preservation, 46(8), 16760 10.1111/jfpp.16760.

[fsn34175-bib-0008] Huang, Y. L. , Chu, Y. L. , Ho, C. T. , Chung, J. G. , Lai, C. I. , Su, Y. C. , Kuo, Y. H. , & Sheen, L. Y. (2015). Antcin K, an active triterpenoid from the fruiting bodies of basswood‐cultivated Antrodia cinnamomea, inhibits metastasis via suppression of integrin‐mediated adhesion, migration, and invasion in human hepatoma cells. Journal of Agricultural and Food Chemistry, 63(18), 4561–4569. 10.1021/jf5059304 25911944

[fsn34175-bib-0009] Irondi, E. A. , Agboola, S. O. , Oboh, G. , Boligon, A. A. , Athayde, M. L. , & Shode, F. O. (2016). Guava leaves polyphenolics‐rich extract inhibits vital enzymes implicated in gout and hypertension in vitro. Journal of Intercultural Ethnopharmacology, 5(2), 122–130. 10.5455/jice.20160321115402 27104032 PMC4835986

[fsn34175-bib-0010] Katsube, T. , Imawaka, N. , Kawano, Y. , Yamazaki, Y. , Shiwaku, K. , & Yamane, Y. (2006). Antioxidant flavonol glycosides in mulberry (*Morus alba* L.) leaves isolated based on LDL antioxidant activity. Food Chemistry, 97(1), 25–31. 10.1016/j.foodchem.2005.03.019

[fsn34175-bib-0011] Kimura, T. , Nakagawa, K. , Kubota, H. , Kojima, Y. , Goto, Y. , Yamagishi, K. , Oita, S. , Oikawa, S. , & Miyazawa, T. (2007). Food‐grade mulberry powder enriched with 1‐deoxynojirimycin suppresses the elevation of postprandial blood glucose in humans. Journal of Agricultural and Food Chemistry, 55(14), 5869–5874. 10.1021/jf062680g 17555327

[fsn34175-bib-0032] Korkmaz, Y. , & Dik, B. (2023). The comparison of the antidiabetic effects of exenatide, empagliflozin, quercetin, and combination of the drugs in type 2 diabetic rats. Fundamental & Clinical Pharmacology. 10.1111/fcp.12975 38149676

[fsn34175-bib-0012] Kreitman, A. , Schneider, S. H. , Hao, L. , Schlussel, Y. , Bello, N. T. , & Shapses, S. A. (2021). Reduced postprandial bone resorption and greater rise in GLP‐1 in overweight and obese individuals after an alpha‐glucosidase inhibitor: A double‐blinded randomized crossover trial. Osteoporosis International, 32(7), 1379–1386. 10.1007/s00198-020-05791-5 33432459

[fsn34175-bib-0033] Kumar, M. , Tomar, M. , Amarowicz, R. , Saurabh, V. , Nair, M. S. , Maheshwari, C. , Sasi, M. , Prajapati, U. , Hasan, M. , Singh, S. , Changan, S. , Prajapat, R. K. , Berwal, M. K. , & Satankar, V. (2021). Guava (*Psidium guajava* L.) leaves: Nutritional composition, phytochemical profile, and health‐promoting bioactivities. Food, 10(4), 752. 10.3390/foods10040752 PMC8066327 33916183

[fsn34175-bib-0013] Lee, J. N. , Kim, H. P. , Hwang, S. Y. , & Chung, W. G. (2002). Single dose toxicity study of Hwangjaegongjinbo, an invigorator, in mice and rats. Journal of Toxicology and Public Health, 18, 73–77.

[fsn34175-bib-0014] Lin, A. H. M. , Lee, B. H. , & Chang, W. J. (2016). Small intestine mucosal alpha‐glucosidase: A missing feature of in vitro starch digestibility. Food Hydrocolloids, 53, 163–171. 10.1016/j.foodhyd.2015.03.002

[fsn34175-bib-0015] Liu, X. M. , Liu, Y. , Shan, C. H. , Yang, X. Q. , Zhang, Q. , Xu, N. , & Song, W. (2022). Effects of five extraction methods on total content, composition, and stability of flavonoids in jujube. Food Chemistry: X, 14, 100287. 35313650 10.1016/j.fochx.2022.100287 PMC8933822

[fsn34175-bib-0016] Medina, N. N. R. , & Herrero, J. V. I. (2016). Guava (*Psidium guajava* L.) cultivars: An important source of nutrients for human health. Nutritional Composition of Fruit Cultivars, 2, 287–315. 10.1016/B978-0-12-408117-8.00013-1

[fsn34175-bib-0017] Naowaboot, J. , Pannangpetch, P. , Kukongviriyapan, V. , Kongyingyoes, B. , & Kukongviriyapan, U. (2009). Antihyperglycemic, antioxidant and antiglycation activities of mulberry leaf extract in streptozotocin‐induced chronic diabetic rats. Plant Foods for Human Nutrition, 64(2), 116–121. 10.1007/s11130-009-0112-5 19434497

[fsn34175-bib-0018] Ojewole, J. A. O. (2005). Hypoglycemic and hypotensive effects of Linn. (Myrtaceae) leaf aqueous extract. Methods and Findings in Experimental and Clinical Pharmacology, 27(10), 689–695. 10.1358/mf.2005.27.10.948917 16395418

[fsn34175-bib-0019] Oki, T. , Matsui, T. , & Osajima, Y. (1999). Inhibitory effect of α‐glucosidase inhibitors varies according to its origin. Journal of Agricultural and Food Chemistry, 47(2), 550–553. 10563931 10.1021/jf980788t

[fsn34175-bib-0020] Ortiz‐Andrade, R. R. , Garcia‐Jimenez, S. , Castillo‐Espana, P. , Ramirez‐Avila, G. , Villalobos‐Molina, R. , & Estrada‐Soto, S. (2007). Alpha‐glucosidase inhibitory activity of the methanolic extract from Tournefortia hartwegiana: An anti‐hyperglycemic agent. Journal of Ethnopharmacology, 109(1), 48–53. 10.1016/j.jep.2006.07.002 16920301

[fsn34175-bib-0021] Shen, N. , Wang, T. F. , Gan, Q. , Liu, S. , Wang, L. , & Jin, B. (2022). Plant flavonoids: Classification, distribution, biosynthesis, and antioxidant activity. Food Chemistry, 383, 132531. 35413752 10.1016/j.foodchem.2022.132531

[fsn34175-bib-0022] Shen, S. C. , Cheng, F. C. , & Wu, N. J. (2008). Effect of guava (*Psidium guajava* Linn.) leaf soluble solids on glucose metabolism in type 2 diabetic rats. Phytotherapy Research, 22(11), 1458–1464. 10.1002/ptr.2476 18819164

[fsn34175-bib-0023] Sun, H. , Saeedi, P. , Karuranga, S. , Pinkepank, M. , Ogurtsova, K. , Duncan, B. B. , Stein, C. , Basit, A. , Chan, J. C. N. , Mbanya, J. C. , Pavkov, M. E. , Ramachandaran, A. , Wild, S. H. , James, S. , Herman, W. H. , Zhang, P. , Bommer, C. , Kuo, S. , Boyko, E. J. , & Magliano, D. J. (2022). IDF diabetes atlas: Global, regional and country‐level diabetes prevalence estimates for 2021 and projections for 2045. Diabetes Research and Clinical Practice, 183, 109119. 10.1016/j.diabres.2021.109119 34879977 PMC11057359

[fsn34175-bib-0024] Sun, Y. , Shao, L. , Niu, X. , Liu, Y. , Ge, J. , Jiang, H. , & Zhang, H. (2014). Clinical effectiveness of Novolin(R) 30R versus Lantus(R) combined with Glucobay(R) treatment in elderly patients with type 2 diabetes mellitus controlled by oral hypoglycaemic agents: A randomized study. The Journal of International Medical Research, 42(4), 993–1001. 10.1177/0300060514529555 24925584

[fsn34175-bib-0025] Vestergaard, P. (2021). Diabetes and its complications – contemporary treatment and potential side effects of drugs to treat diabetes. Current Drug Safety, 16(1), 2. 33726642 10.2174/157488631601210203093615

[fsn34175-bib-0026] Wu, J. W. , Hsieh, C. L. , Wang, H. Y. , & Chen, H. Y. (2009). Inhibitory effects of guava (*Psidium guajava* L.) leaf extracts and its active compounds on the glycation process of protein. Food Chemistry, 113(1), 78–84. 10.1016/j.foodchem.2008.07.025

[fsn34175-bib-0027] Yang, J. B. , Tian, J. Y. , Dai, Z. , Ye, F. , Ma, S. C. , & Wang, A. G. (2017). A‐glucosidase inhibitors extracted from the roots of Polygonum multiflorum Thunb. Fitoterapia, 117, 65–70. 10.1016/j.fitote.2016.11.009 27889542

[fsn34175-bib-0028] Yousaf, A. A. , Abbasi, K. S. , Ahmad, A. , Hassan, I. , Sohail, A. , Qayyum, A. , & Akram, M. A. (2021). Physico‐chemical and nutraceutical characterization of selected indigenous guava (*Psidium guajava* L.) cultivars. Food Science and Technology, 41(1), 47–58. 10.1590/fst.35319

[fsn34175-bib-0029] Zhang, M. , Chen, M. , Zhang, H. Q. , Sun, S. , Xia, B. , & Wu, F. H. (2009). In vivo hypoglycemic effects of phenolics from the root bark of. Fitoterapia, 80(8), 475–477. 10.1016/j.fitote.2009.06.009 19545615

